# Clinical and Pathological Variation of Charcot-Marie-Tooth 1A in a Large Chinese Cohort

**DOI:** 10.1155/2017/6481367

**Published:** 2017-08-01

**Authors:** Rui Wu, He Lv, Wei Zhang, Zhaoxia Wang, Yuehuan Zuo, Jing Liu, Yun Yuan

**Affiliations:** Department of Neurology, Peking University First Hospital, Beijing 100034, China

## Abstract

Charcot-Marie-Tooth 1A (CMT1A) caused by peripheral myelin protein 22 (*PMP22*) gene duplication is the most common form of hereditary polyneuropathy. Twenty-four genetically confirmed CMT1A patients with sural nerve biopsies were enrolled in this study. The clinical picture included a great variability of phenotype with mean onset age of 22.2 ± 14.5 years (1–55 years). Pathologically, we observed a severe reduction in myelinated fiber density showing three types of changes: pure onion bulb formation in 3 cases (12.5%), onion bulb formation with axonal sprouts in 10 cases (41.7%), and focally thickened myelin with onion bulb formation or/and axonal sprouts in 11 cases (45.8%). We observed no significant correlation between nerve fiber density and disease duration. There was no significant difference between the 3 pathological types in terms of clinical manifestations, nerve fiber density, and *g*-ratio. Our study indicates that there is marked variability in the age of onset of CMT1A, as well as significant pathological changes without deterioration with the development of the disease. Focally thickened myelin is another common morphological feature of demyelination.

## 1. Introduction

Charcot-Marie-Tooth (CMT) disease, the most common hereditary sensorimotor neuropathy, has a prevalence of 1 : 2,500 [1]. CMT1A is caused by duplication of the peripheral myelin protein 22 (*PMP22*) gene accounting for more than half of all the CMT cases. The disease usually occurs in the second and third decades [2], with slowly progressive length-dependent neuropathy [3, 4]. Dejerine-Sottas syndrome [5, 6], or onset in the late adulthood, has been described occasionally. The motor nerve conduction velocity (MNCV) in median nerves reduces severely [2, 7] but slightly increases with the age of MNCV, potentially due to myelin thickness remodeling that occurs later in CMT1A patients [8]. Previous studies have shown that the compound muscle action potential amplitude (CMAP) was reduced from an early age, and its typical increase with age was attenuated [9]. The clinical severity of the disease at the impairment and disability levels was related to the degree of axonal dysfunction [10]. Pathologically, sural nerve biopsies revealed severe reduction in myelinated fiber density and high percentage of onion bulb formations [11]. A preferential distal axonal loss, associated with axonal atrophy, degeneration, and axonal sprouting, was observed [10, 12]. Recently, tomacula or hypermyelination of myelinated fibers appeared in patients with* PMP22* duplication [13]. So, nerve biopsy can show different typical pathological changes in CMT1A which plays an important role in diagnosing this disease despite the wide use of genetic testing. Here, we describe the pathological features in a large cohort of Chinese patients with CMT1A.

## 2. Materials and Methods

Ninety-two probands of CMT were diagnosed by next generation sequencing and traditional DNA sequencing at the Department of Neurology, Peking University First Hospital, from January 2005 to January 2016. Eighty-seven probands (94.6%) had a pattern of inheritance compatible with autosomal dominant transmission. Twenty-four probands from unrelated families identified with the* PMP22* gene duplication therefore were diagnosed with CMT1A and recruited in this study (Table 1), which made up 26.1% of the total CMT families. This study was approved by the Health Authority Ethical Committee of Peking University First Hospital. All participants provided written informed consent. Patients' histories were collected. Nerve conduction studies were performed in 14 patients.

Sural nerve biopsies were performed in all cases. Nerve specimen was processed using standard methods as follows: one specimen was fixed in 4% formalin, embedded in paraffin, and stained with hematoxylin-eosin, Luxol Fast Blue, and Congo Red. Another was fixed in 2-3% glutaraldehyde and postfixed in 1% osmium tetroxide. Semithin sections for light microscopy were stained with Toluidine Blue. Ultrathin sections were contrasted with uranyl acetate and lead citrate and then examined under electron microscopy. The nerve fiber density was calculated using NIS-Elements BR 3.2 program. Thick or thin myelin sheath encircled can be confirmed by the *g*-ratio, which was calculated from EM photographs of at least 30 fibers (9000x final magnification). The pathological changes of myelinated fibers were divided into 3 groups: (1) pure onion bulb formation, (2) onion bulb formation with axonal sprouts, and (3) focally thickened myelin with onion bulb structures or axonal sprouts.

The statistical analyses were performed using SPSS23.0. Pearson's correlation coefficient (*r*) was used to determine the correlation between nerve fiber density, the degree of muscle weakness, the age of onset, and disease duration. The age of onset, disease duration, degree of muscle weakness, sensory loss, foot deformity, median nerve MCV, median nerve CMAP, nerve fiber density, and *g*-ratio were compared in different pathological subtypes by the variance analysis. Probability values of *P* < 0.05 were considered statistically significant.

## 3. Results

**Figure 1:**
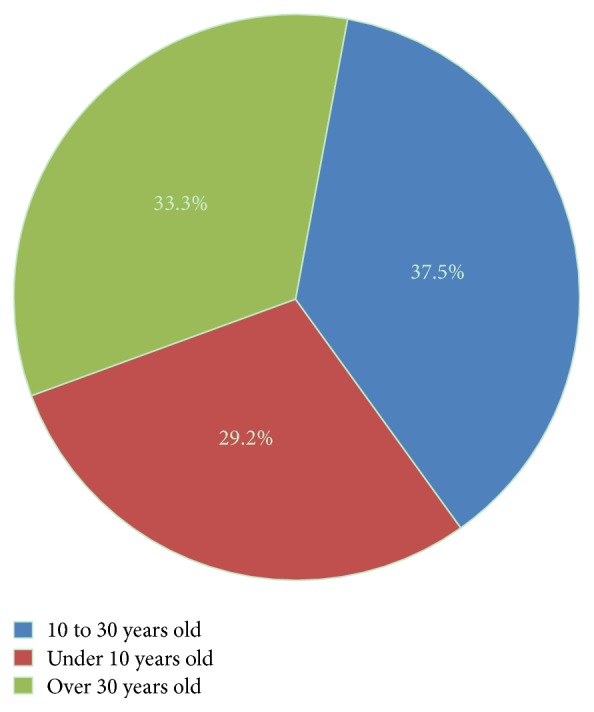
Composition of CMT1A patients with different ages of onset.

From the 24 CMT1A patients, 15 (62.5%) were males and 9 (37.5%) were females. The average age of onset was 22.2 ± 14.5 years (1–55 years), including seven patients (29.2%) with an early onset before the age of 10 years (one patient with congenital onset after birth), nine classical patients (37.5%) between the ages of 10 and 30 years, and eight patients with a late onset after the age of 30 years (33.3%) (Figure 1). The mean disease duration between onset and diagnosis time was 7.2 ± 6.5 years (0.5–26 years). The main symptoms were steppage gait in 20 cases (83.3%), with myalgia in legs in three patients (12.5%). Neurological examination showed distal weakness of legs in 20 cases (83.3%), accompanied with distal weakness with muscle atrophy in the upper limbs in 13 cases (54.2%), calf atrophy in 10 patients (41.7%), sensory loss in distal limbs in 15 patients (62.5%), absent deep tendon reflexes in 22 patients (91.7%), and pes cavus in 13 cases (54.2%). Four patients (16.7%) showed no muscle weakness or sensory loss in all limbs.

The MNCV was 18.6 ± 12.5 m/s (0–51.0 m/s) in the median nerves and was slower than 38 m/s in 13 cases. Eleven cases had decreased amplitude of the CMAP and three cases showed no CMAP.

Sural nerve biopsy showed that the mean nerve fiber density was 3269.4 ± 1989.3/mm^2^ (1022.0–7390.0/mm^2^). The mean *g*-ratio was 0.47 ± 0.06 (0.31–0.57). The myelin sheaths were normal with no evidence of decompaction or redundant loops. Nine patients had thickened capillary basal membrane (37.5%). Three patients had edema in fascicules (12.5%).

**Figure 2:**
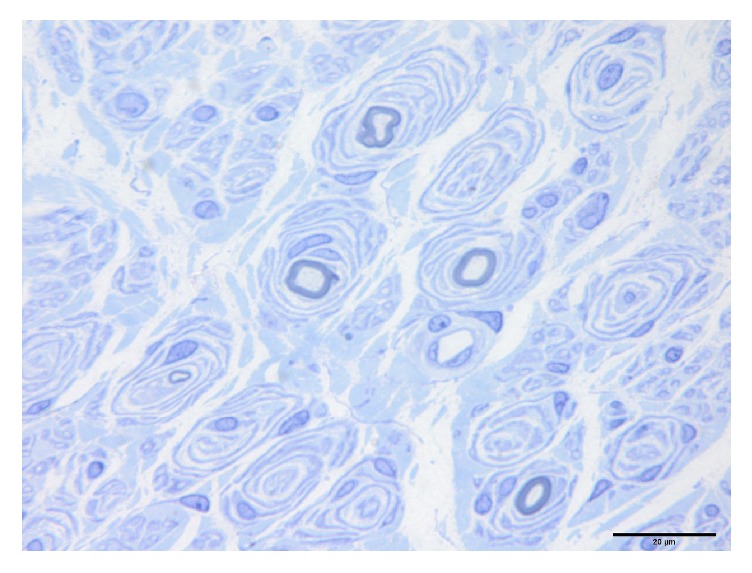
Sural nerve biopsy of a demyelination dominant CMT1A patient showing features of diffuse onion bulb lesions.

**Figure 3:**
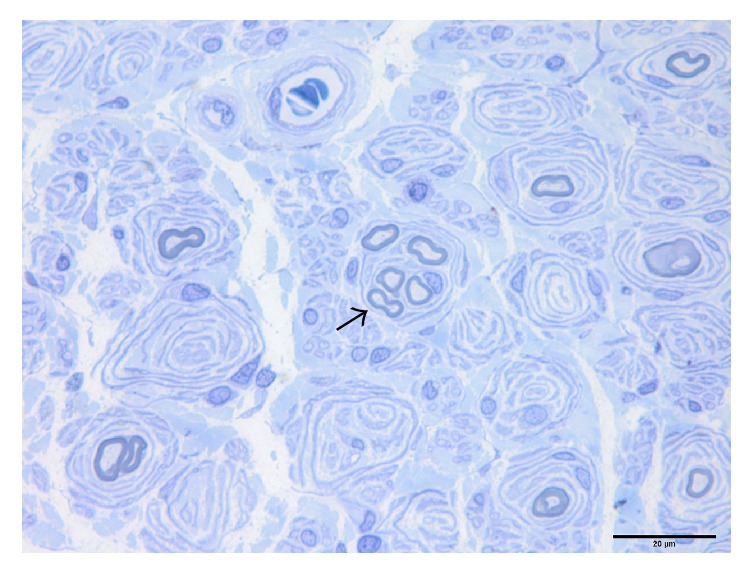
Sural nerve biopsy of a mixed myelin-axonal damage CMT1A patient showing features of typical onion bulb lesions and regeneration clusters (black arrow).

**Figure 4:**
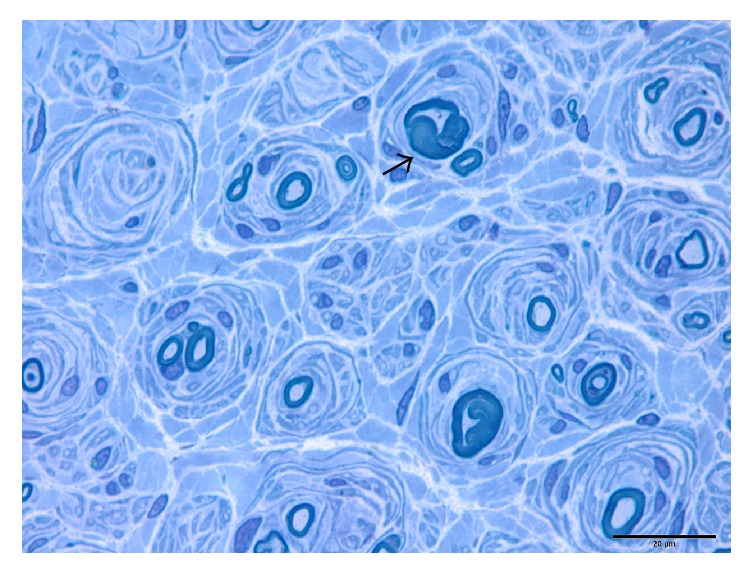
Sural nerve biopsy of a CMT1A patient showing features of focally thickened myelin (black arrow).

All these 24 cases can be divided into three subtypes based on their pathological changes shown in Table 2. Pure onion bulb formation (Subtype 1) was observed in 3 cases (12.5%). These patients had numerous onion bulb structures (Figure 2). The mean nerve fiber density was 2167.7 ± 107.7/mm^2^ (2045.0–2247.3/mm^2^). The mean *g*-ratio was 0.40 ± 0.06 (0.35–0.46). Onion bulb formation with axonal sprouts (Subtype 2) was seen in 10 cases (41.7%) (Figure 3). There were 2 to 4 small myelinated fibers in a cluster. The mean nerve fiber density was 3746.1 ± 1937.6/mm^2^ (1837.1–6842.2/mm^2^). The mean *g*-ratio was 0.42 ± 0.06 (0.37–0.54). Focally thickened myelin (Subtype 3) was seen in 11 cases (45.8%) with onion bulb structures or axonal sprouts (Figure 4). The distribution of focally thickened myelin was isolated from onion bulb formation or axonal sprouts. The mean nerve fiber density was 3144.3 ± 1858.8/mm^2^ (1022.3–7390.0/mm^2^). The mean *g*-ratio was 0.44 ± 0.08 (0.31–0.57).

Statistical analysis showed that there was no significant correlation between nerve fiber density, the degree of distal muscle weakness of legs (*r*^2^ = 0.04, *P* = 0.39), the age of onset (*r*^2^ = 0.04, *P* = 0.36), and disease duration (*r*^2^ = 0.06, *P* = 0.26). The MNCV and CMAP of median nerve were not correlated with the age of onset, disease duration, and fiber density.

There was no significant difference between the 3 pathological types in terms of gender (*F* = 0.02, *P* = 0.98), age of onset (*F* = 0.10, *P* = 0.91), disease duration (*F* = 0.73, *P* = 0.49), degree of distal weakness of legs (*F* = 0.08, *P* = 0.93), median nerve MCV (*F* = 0.87, *P* = 0.45), median nerve CMAP (*F* = 3.6, *P* = 0.07), fiber density (*F* = 0.83, *P* = 0.45), and *g*-ratio (*F* = 0.54, *P* = 0.59). Patients with thickened capillary basal membrane had later onset (25.3 ± 11.5 years) compared to those with normal capillary basal membrane (12.5 ± 15.4 years) (*t* = 2.15, *P* = 0.04).

## 4. Discussion

In the present series of Chinese CMTs from our department, autosomal dominant CMT (AD-CMT) is more frequent than autosomal recessive CMT (AR-CMT). AD-CMT1A was identified in 24 (26.1%) of the total 92 CMT families. This is more common compared to 13.6% (20/148) in a study based in southern China [14]. The mean age of onset is 22.2 years old in the present series, while it was 15.5 years old in Korean patients [15]. The age of onset after 30 years is more common than previously reported [16].

The main neuropathological change in this cohort of AD-CMT1A is loss of myelinated fiber which is a result of apoptosis of Schwann cells because of overexpressed PMP22, with Schwann cell proliferation forming onion bulbs and demyelination. These features have also been reported by others [11, 13]. Regeneration cluster of myelinated fibers is another common change in this cohort, indicating that pathologically AD-CMT1A is associated with axonal degeneration that is secondary to demyelination [12, 17]. Focally thickened myelin can be seen in nearly half of our patients which is another sign of demyelination. This may be a sign of PMP22 aggregation due to overexpressed PMP22 overwhelming the protein degradation system. Therefore, three types were suggested based on the pathological changes of myelinated fibers: pure onion bulb formation, onion bulb formation with axonal sprouts, and focally thickened myelin with onion bulb formation or/and axonal sprouts. Pathological features are essential for the diagnosis of different types of CMT1A as well as further study of its pathogenesis, which may contribute to the development of new treatments.

Electrophysiological study suggested that the clinical disabilities of CMT1A are determined by the extent of axonal dysfunction [15]. Myelinated fiber densities did not decrease with the duration of the disease, and there was no difference between the three pathological subtypes. Only the thickened capillary basal membrane existed in the late onset of these patients, which might be an age-related change [18]. With the exception of one case with congenital hypomyelination neuropathy, *g*-ratio value is normal in other patients. In contrast to a previous report that indicated that patients under 15 years old had lower *g*-ratio values [19], we did not find a statistically significant relationship between the age of onset and *g*-ratio values. Clinically, patients with lower muscle strength did not have less nerve fiber density. The severity of neurologic deficits and slowing of MNCV in the median nerve did not vary significantly with the age of patients [19]. Our result supported the notion that pathological changes formed in the early stage of CMT1A patients and progressed little during the course of the disease.

Onion bulb formation is the most common change in CMT1A [10–13]. However, there were only a few cases with pure onion bulb formation, mostly combined with axonal sprouts or focally thickened myelin. Onion bulb formations were distributed diffusely. The patchy distribution of onion bulb formation was reported in Japanese patients [11], which was not observed in our study. The fiber density was lower in patients with pure onion bulb formation compared with those with axonal sprouts or focally thickened myelin. Nevertheless, there is no significant difference among the three pathological subtypes. The nonsignificance of these results may be due to a smaller sample size.

Focally thickened myelin indicates demyelination and is another common pathological change in CMT1A. Thickened myelin-like tomacula present in pressure palsy are the result of* PMP22* deletion [13]. It is considered as partial tomacula and rarely appears in onion bulb formation. The group of focal thickened myelin shows lower *g*-ratio. However, the difference is not significant between patients with and without thickened myelin.

In summary, there is a marked variability in the age of onset of CMT1A. However, the pathological process does not deteriorate with the development of the disease.* PMP22* duplication leads to low density of myelinated fibers with different pathological changes. In addition to onion bulb formation, focally thickened myelin is another common morphological feature for demyelination. Axonal degeneration or regeneration is not rare.

## Figures and Tables

**Table 1:** Clinical features and nerve conduction studies (NCS) of the patients analyzed in this study.

Patient number	Gender	Age of evaluation (years)	Age of onset (years)	Disease duration (years)	Strength of muscle		Pes cavus	Sensory loss	Areflexia	Muscle atrophy		Median nerve MCV (m/s)	Median nerve CMAP (mV)
					Upper limb	Lower limb				Hand	Calf		
1	F	13	7	6	5	5	Y	Y	Y	Y	N	21.0	3.3
2	M	8	8	0.5	4	4	N	N	Y	N	N	—	—
3	M	27	26	1	5	2	Y	Y	Y	N	N	—	—
4	M	37	11	26	4	2	Y	N	Y	N	N	11.0	1.9
5	M	32	24	8	4	3	N	Y	Y	Y	Y	51.0	4.6
6	M	39	32	7	4	4	N	Y	Y	Y	Y	—	—
7	F	48	39	9	4	2	N	N	Y	Y	Y	—	—
8	F	45	25	20	5	4	N	Y	Y	Y	Y	—	—
9	F	9	5	3	5	4	Y	Y	Y	N	N	14.0	5
10	F	38	36	2	3	2	Y	Y	N	Y	N	14.0	—
11	F	28	13	15	4	4	Y	Y	N	Y	N	0	0
12	F	36	33	3	4	4	Y	N	Y	Y	N	20.0	1.3
13	M	8	1	7	4	4	Y	N	Y	N	N	30.0	5
14	M	33	23	10	3	3	N	N	Y	Y	Y	—	—
15	M	9	6	3	5	5	Y	Y	Y	Y	Y	—	—
16	M	59	55	4	5	4	N	Y	Y	N	N	22.0	2.4
17	M	21	2	18	4	1	Y	Y	Y	N	Y	22.1	7.9
18	M	31	30	1	5	5	Y	N	Y	N	N	—	—
19	M	42	39	3	4	4	Y	Y	Y	Y	Y	24.0	—
20	M	35	31	4	5	4	Y	Y	Y	Y	Y	—	—
21	F	36	32	4	5	3	N	N	Y	N	N	16.0	2.4
22	F	35	25	10	4	1	N	Y	Y	Y	Y	15.9	1
23	M	31	28	3	5	5	N	Y	Y	N	N	0	0
24	F	8	1.5	6	5	4	N	N	Y	N	N	—	—

*Note*. M: male; F: female; Y: yes; N: no; MCV: motor conduction velocity; CMAP: compound muscle action potential amplitude. Black bar means material defect.

**Table 2:** Comparison of clinical characteristics between three different pathological CMT1A subtypes.

	Subtype 1	Subtype 2	Subtype 3
Number of patients	3	10	11
Age at onset (years)	19.7 ± 11.9	21.5 ± 12.1	23.5 ± 17.8^*※*^
Age at examination (years)	25.0 ± 14.0	30.6 ± 12.7	29.7 ± 16.3^*※*^
Disease duration (years)	5.3 ± 4.0	9.2 ± 8.4	6.0 ± 5.0^*※*^
Distal muscle weakness			
Upper limbs	4.7 ± 0.6	4.2 ± 0.6	4.5 ± 0.7^*※*^
Lower limbs	3.7 ± 2.3	3.5 ± 0.8	3.4 ± 1.3^*※*^
Muscle atrophy			
Hand	2 (66.7%)	7 (70%)	4 (36.4%)^*※*^
Calf	2 (66.7%)	4 (40%)	4 (36.4%)^*※*^
Sensory loss	3 (100%)	6 (60%)	6 (54.5%)^*※*^
Areflexia	3 (100%)	8 (80%)	11 (100%)^*※*^
Foot deformity	1 (33.3%)	5 (50%)	7 (63.6%)^*※*^

Subtype 1: pure onion bulb formation; Subtype 2: onion bulb formation with axonal sprouts; Subtype 3: focally thickened myelin with onion bulb formation or/and axonal sprouts. ^*※*^*P* > 0.05.
